# When the phone rings - factors influencing its impact on the experience of patients and healthcare workers during primary care consultation: a qualitative study

**DOI:** 10.1186/s12875-015-0330-x

**Published:** 2015-09-02

**Authors:** A. Y. L. Koong, D. Koot, S. K. Eng, A. Purani, A. Yusoff, C. C. Goh, S. S. H. Teo, N. C. Tan

**Affiliations:** SingHealth Polyclinics, 167 Jalan Bukit Merah #15–10, Singapore, 150167 Singapore; Choa Chu Kang Family clinic, Blk 304, Choa Chu Kang Ave 4 #01–653, Singapore, 680304 Singapore; Duke-NUS Graduate Medical School, Singapore, Singapore

## Abstract

**Background:**

In the primary health care setting, patients interact directly with their healthcare workers (HCW), which include their primary physicians, nurses and pharmacists. Studies have shown that such interactions, when interrupted by phone calls received by either party, can lead to adverse outcomes and negative experiences. There is insufficient data however on the factors affecting the reaction and responses of both patients and HCWs when phone calls occur amidst their interaction. Understanding these factors will allow for the introduction of targeted measures to mitigate the negative impact of such interruptions and improve patient-HCW relationships. This study therefore aims to understand the impact of unplanned phone calls during primary health care consultations on patient–HCW interactions and the factors affecting the patient and the HCW responses.

**Method:**

This study used focus group discussions (FGD) to gather qualitative data from patients and HCWs who had visited or worked in a major public primary healthcare institution in Singapore. The FGDs were audio-recorded, transcribed, audited and analyzed using standard content analysis to identify emergent themes.

**Results:**

15 patients and 16 HCWs participated in 5 FGDs. The key themes that emerged from these FGDs were patients’ and HCWs’ attitudes toward professionalism and respect, task and thought interruption, call characteristics, the impact on patient safety and stakeholders’ experiences. Phone calls during consultations answered by either party often resulted in the answering party feeling apologetic and would usually keep the phone conversations short as a sign of respect to the other party. Both stakeholders valued the consultation time and similarly reported negative experiences if the phone-call interruptions became prolonged. Calls from the desk phone answered by HCWs were perceived by most patients to be relevant to healthcare services, with the assumption that HCWs exercised professionalism and would not attend to personal calls during their clinical duties.HCWs expressed their concerns and distress about potential medical errors due to phone-calls interrupting their clinical tasks and thinking processes. However, they acknowledged that these same phone-calls were important to allow clarifications of instructions and improved the safety of other patients.

**Conclusion:**

Phone interruptions affected patient and HCW interaction during consultations and factors leading to their adverse reactions need to be recognized and addressed.

## Background

Interruptions are common during primary care consultations [1–4]. Chisholm et el reported that primary care physicians were interrupted at an average rate of 3.9 times per hour [1]. Other studies revealed that interruptions affected up to 94 % [2–4] of primary care consultations. While such interruptions were not limited to primary care settings, they affected the work of healthcare workers (HCW) such as nurses and pharmacists [5–8].

Interruptions have various effects such as increased length of time to perform a task [9, 10] and contributed to errors involving prescriptions [11], administration and dispensing of medication [5–7]. Dearden et al found that interruptions adversely affected one in five patients while 40 % would have preferred that their consultations were not interrupted [2]. In experimental studies, participants subjected to interruptions while performing tasks experienced increased stress, annoyance and frustration [10, 12]. A qualitative study reported that interruptions increased the risk of errors due to distractions and interrupted thought processes in primary care [11]. It was also one of the causes of job stress reported amongst general practitioners [13].

The telephone accounted for about 14–50 % of the interruptions [2–4]. In one study, the top three causes of interruption were phone calls for the patients, followed by phone calls for the HCW and lastly knocks on the door [3]. In a study conducted in Colombia, about 8 to 13 % of patients received or made at least 1 call, averaging 21 seconds, during the consultation. A lower proportion of patients did so when a printed reminder was placed in the waiting room [14]. Whilst it is common to find regulation of phone use in the hospitals, guidelines are generally lacking in the primary care clinics.

In Singapore, phone calls occur commonly during medical consultations, especially in the primary care setting. SingHealth Polyclinics (SHP), one of two local clusters of 9 public polyclinics, managed more than 1.7 million patient attendances in 2014. This institution uses an electronic clinical health record and prescription system and has 1030 multi-disciplinary staff. Calls are often necessary for patient care and inter-disciplinary communication. Singapore has also one of the highest local population mobile phone penetration rate (150 % in 2012), hence one can often see HCWs and patients in SHP using their personal mobile phones or landlines for communication.

We chose to study the effect of phone-calls as the main source of interruption, as it commonly occurred to both patient and HCW. Such interruptions have been reported by HCWs in our primary care setting to be associated with prescription errors and negative emotions such as frustration. With the large number of primary care encounters in our practice, as well as the high rate of phone usage, we postulate that the occurrence of unplanned phone calls during the primary care consultations would be inevitable. However, the impact on the experience of the patient and the HCW is not well understood as there are variable factors associated with each unique patient and HCW encounter. The interplay of these factors would influence the experience of both parties during such interrupted consultations.

An in-depth understanding of these factors, and experience of the involved stakeholders could guide healthcare institutions in designing measures to mitigate the negative impact of phone call interruption and improve the experience of patient and HCW during primary care consultations.

## Methods

Qualitative data was gathered using focus group discussions (FGD), which involved patients as well as HCWs. An exploratory approach, set within the grounded theory framework [15], was chosen as little data is available on the topic in the local healthcare setting in Singapore. The scenarios could be potentially complex, with different categories of HCWs affected, as well as with different characteristics of the interrupting phone calls. A qualitative research method would therefore be an appropriate way to gain in-depth understanding of the interplay of factors affecting the experience of the patients and HCWs in different contexts. This study was approved by the SingHealth Centralised Institutional Review Board (CIRB reference 2012/299/E) and received seed funding from SingHealth Polyclinics.

Patients were approached by investigators at various service points in the clinic and asked verbally if they had experienced a phone call during a medical consultation either currently or in the past. Patients who gave a positive response were then invited to participate in a FGD. Snow-balling method was also used in the recruitment process, where recruited participants were encouraged to attend the FGD with friends or family who had similar experiences with regards to phone interruptions during a consultation. Patients who had cognitive impairment or who were not able to communicate in English were excluded.

Electronic invites were sent via e-mails to HCWs, comprising of physicians, nurses and pharmacists inviting them to participate voluntarily in the FGDs if they had previously experienced a phone call interruption during a primary care consultation. These interrupted encounters were not required to fall within a specified period as the investigators felt that there could be recall bias of the occurrence of such encounters leading to inaccurate data.

Potential participants were provided with study information leaflets detailing the study’s objective, method, schedule and sites for the FGDs. In order to create variability in participant characteristics such as age, race, and gender, invitations to these participants were sent out in stages. Each FGD would thus have patients and HCWs with different profiles. All participants of the FGDs were reimbursed a small token sum for their transport expenses.

Written informed consent was obtained from the participants prior to the commencement of the FGDs, during which their anonymity was maintained using coded identities. Assigned investigators moderated the FGDs, which were conducted in English. The moderator used an approved topic guide, which covered the following topics: participants’ views on the use of phones during consultation, examples of phone usage during the primary care consultation, as well as the experience of the patients and HCWs as a result of the interrupted primary care consultation. All interviews were audio-recorded and assigned transcribers carried out verbatim transcription. These transcripts were checked for accuracy against the original audio-recordings of the FGDs by designated investigators in the team.

The principle investigator and co-investigator analyzed the transcripts separately, and the initial codes were medical errors, disruption of tasks and thought processes, emotions and reactions, mutual respect, professionalism and call characteristics. These initial codes led to the observation of major themes such as safety, attitude towards the patient and HCW relationship, experience of the patient and HCW as well as call characteristics. Themes appeared to be saturated by the 3^rd^ FGD and no new codes were found. Two additional FGDs were carried out to ensure that no new themes emerged. As the codes and themes could be influenced by the investigator’s experience of interrupted consults, the two investigators attempted to reduce the bias of analysis by triangulating their findings and discussed the interactions between themes. These interactions are described in the next section.

## Results

A total of 31 participants were interviewed in 5 FGDs between September 2012 and June 2013. The demographic characteristics of the participants are shown in Table 1. The 3^rd^ FGD was organized to include a mix group of participants comprising of both patients and HCW so as to observe their interaction and exchange of views on topics discussed during the FGD. The stakeholders included the HCW and patients.

**Table 1:** Demographic characteristics of participants

	FGD 1	FGD 2	FGD 3	FGD 4	FGD 5	Total
Number of Participants						31
Patient	8	0	2	0	5	15
Doctor	0	3	1	2	0	6
Nurse	0	2	5	1	0	8
Pharmacist	0	1	0	1	0	2
Gender						
Male	2	3	0	3	4	12
Female	6	3	8	1	1	19
Age (years)						
<30	0	0	2	0	0	2
30–60	8	6	6	4	0	24
>60	0	0	0	0	5	5
Ethnic groups						
Chinese	7	6	3	3	4	23
Malay	0	0	4	0	0	4
Indian	0	0	0	1	1	2
Others	1	0	1	0	0	2

The following themes emerged, relating to the impact of phone interruptions on stakeholders’ attitude towards professionalism and mutual respect, patient safety, experience and efficiency of healthcare service delivery.

### Professionalism and mutual respect

The stakeholders’ attitude towards professionalism and mutual respect influenced their experience of the interrupted consultation. Patients expected doctors to exercise professionalism by not answering calls that were personal or unrelated to patient care during the consultation. HCW reported that they would voluntarily take measures to avoid interruptions from personal calls during consultation. Some patients reported that they would likewise do the same as a sign of respect. These measures included turning off their mobile device, setting the devices to silent mode, not answering the calls or keeping the calls short. They were apologetic when they took the call in the middle of an encounter. Such attitude and behavior appeared to mediate the expectation and experience of either stakeholder when their interaction was interrupted by an unplanned phone-call.

“*If the call is not important, you expect them to just turn off or say sorry, I am busy now. So at least you can carry on with the meeting. I mean that’s what we do in the corporate meetings. So since doctors are professionals, we expect the same behavior*.” Patient P8, FGD2

“*I usually put it (my handphone) on silent mode during consultation.”* Doctor H6, FGD1

“*As far as I am concerned, if I am consulting the doctor, I have to respect the doctor, I will let the other party know that I will return the call later*.” Patient P14, FGD5

“*If it (personal mobile phone) does vibrate or ring, if I have forgotten to turn it off, I will apologize to the patient and I will end the call. I would not answer it in front of the patient.*” Doctor H6, FGD1

Patients and HCW perceived calls coming through the landline to be related to the HCW’s professional duties, and were deemed important. Patients could understand that these calls were unavoidable, as communications was often necessary between HCWs in the delivery of healthcare services and would accept the interrupted consultation.

*“If you are receiving a call from the desk phone, I think patients would be more understanding ‘cause they can tell that it’s work related.”* Patient P3, FGD2

Just as patients perceived that taking work-related calls were important and part of the job, HCW acknowledged that some calls could be important to patients, and would likewise be agreeable to their patients taking the call.

*“I have people who are in the business world, expatriates and high-ranking people. Some of the calls are very urgent, very important, they have to answer. … They will speak a little and they will tell the caller that they will call back …can be an overseas call, thus very important.”* Doctor H20, FGD4

*“I have patients using hand phones during the consult but overall I think it’s not very disruptive, because …they might have some important thing, so they have to answer the call but they tend not to chit chat on; they tend to just say the relevant things and then put (it) down.”* Nurse H3, FGD1

With regards to professionalism, HCW described feeling stressed when the phone rang in the midst of performing a procedure or physical examination, during which they felt compelled but were unable to answer the call.

“*You feel really stressed over the phone ringing or if you’re doing some procedure or examination and then the phone is ringing away non-stop. You’re stuck because you know that there’s something that can potentially be an emergency*…” Doctor H6, FGD1

HCW were irked when patients disregarded the consultation and continued with their phone conversation.

“*The patient who, once in a while, answers the call with no deference to me…talks a long time while I am waiting in the consult room…valuable time being lapsed….these are the patients who irritate us. They don’t even give us warning that the call is coming through and will take a long time*.” Doctor H20, FGD4

### Patient safety

Phone calls that caused task and thought interruption were perceived by HCWs to increase the risk of medical errors. HCWs were concerned about the negative impact on patient safety and cited examples of these errors when the phone call interrupted the HCW in the midst of executing a primary task. When HCWs were interrupted by phone calls, they reported opportunities for errors, such as switching to tasks involving other patients besides the one they were serving. This was especially so in a situation where there was high cognition demand on the HCW. HCWs gave examples of such situations where near misses had occurred, such as during medication prescription, medication dispensing and immunization. Patients described similar experience of failure to complete the intended task as a result of thought interruptions.

*“… the pharmacist calls you (by phone to inquire) about a particular patient. You switch the electronic medical record to that (previous) patient to check the prescription and make the amendment. Then you have to re-engage the patient right in front of you, and if you are not absolutely conscious about this switch, you could end up prescribing under the wrong identity. These are near misses that have happened before. We have to recognize this: when we try to multitask, especially in a very busy clinic, mistake inevitably will happen.”* Doctor H19, FGD4

“*I think it is quite disruptive… you are doing work and you have a certain line of thought. You are already multi-tasking, so when the phone rings, you have to answer and it can actually cause you to lose your thoughts*.” Pharmacist H17, FGD4

“*The problem is… I wanted to relate something but was interrupted. When I stop for too long, I will have forgotten what I have wanted to say*.” Patient P12, FGD5

Stress was once again described as a negative emotion when HCWs experienced thought interruption as a result of incoming phone -calls.

*“I mean you get stressed up ….when the train of thought is disrupted, you get a bit disorganized and when you get disorganized, your momentum fails and then it just prolongs the consultation period. The burden is ours … while we don’t show our stress to the patient. It is painful!”* Nurse H3, FGD1

HCW described situations where incoming phone calls meant for the patients’ accompanying care-givers affected them as well.

“*(In) the immunization room where you have to do your procedure in a very systematic way; the parent takes a call, you have to stop, and once you stop, the (work) flow gets disrupted and I will get irritated. The danger is that you may forget something or something has changed*.” Nurse H3, FGD1

Phone interruptions presented other opportunity for errors, such as those, which disrupted the patient-HCW interaction and allowed a second patient to engage the HCW during the period of time in which the first patient was attending to a phone call.

***“****When other people see this patient in front of me chit chatting (over the phone), they will try to come and interrupt….so if that is the case, we can make mistakes and yes, there will be a danger!*” Pharmacist H1, FGD1

Patients shared their concern about receiving inaccurate instructions from their provider as a result of the phone-call, which disrupted the communication process.

*“I think the patient might feel a bit insecure here … (when) he (the doctor) got distracted halfway (by phone call); so can I be sure that his instructions are correct? So I might double check with the nurse again … yeah, so there is the insecurity there.”* Patient P3, FGD2

HCWs appeared to be aware of the risk of errors associated with a disrupted work or thought process. Hence they developed negative emotions such as stress, which negated their experience of the encounter. Patients on the other hand did not appear to be adversely affected. This could point to a lack of awareness of the risk of interruption and its impact on the safety of the care delivered. This will be an important area to address.

If the phone call happened during a time when task substitution was possible, the HCW seemed less bothered. Under these circumstances, the HCW could carry on with other tasks while the patient was engaged on the phone, minimizing time wastage.

“*I don't mind because I take that span of time to collect my thoughts. Normally, I need to collect my thoughts or key in more information into the clinical document. I feel more irritated when it’s my phone that rings …if it is the patient who answers his own phone, I can still continue own train of thoughts. Most of the time, I have something else to do anyway, especially towards the end of the consult. I am printing the medication prescription or printing MC (medical certificate)… so it doesn’t really matter that they are talking (over the phone).”* Doctor H11, FGD3

“*When I dress the wound, it’s fine with me even if they are on the phone.”* Nurse H12, FGD3

Ironically some of the phone calls that interrupted the patient HCW encounter could be for the purpose of prescription clarification, which allowed rectification and prevention of a prescription error. HCWs were more appreciative of such calls, which helped to improve patient safety.

“*Most of the time, it is called for and even at times, it is to my benefit because there was indeed a prescription problem. So most of the time, I appreciate the call (from the pharmacy)*.” Doctor H 4, FGD1

*“(Calls) actually help clarify certain things … that may otherwise lead to some error, which is even more serious!”* Pharmacist H17, FGD4

### Experience and efficiency of health service delivery

The content and context of the phone call affected the efficiency of the health service delivery and the consultation experience. Many patients and HCWs valued the limited consultation time in the busy clinic setting and felt that the efficiency and quality of care delivered could be hampered by phone interruptions, especially if the calls were perceived to be irrelevant or lengthy. Some HCWs expressed their frustration when dealing with such encounters.

*“You are coming for a consultation and I think you should remain one to one and there should be no-interference or interruption.”* Patient P6, FGD2

“*Our work is very intense in primary care because we need to take relevant history in the shortest possible time. Most of the consultations take only about 5–8 min, so if your call takes about 1 min, that’s like almost 10 % of the consult time. It’s important to us because we can take relevant history during that time…*” Doctor H2, FGD1

*“Majority of the phone calls are short, but once in a while, we do encounter the business woman coming in, taking orders over the phone during the consultation….that really works us up.”* Nurse H13, FGD4

***“****It’s frustrating! A lot of them (referring to phone calls) are (from) the bank callers and the insurance companies*.” Doctor H4, FGD1

The HCWs frowned upon inconsiderate behavior where patients being served would hold up the consultation by attending to phone calls. Such interruptions would prolong the waiting time of other patients in the queue.

*“If we are at the counter where there are lots of patients waiting to be served, and if you are still on the phone and I can tell that you are just chit-chatting away, you are wasting my time and wasting other patient’s time, so that does get me irritated.”* Pharmacist H1, FGD1

## Discussion

In our practice, phone calls occur commonly during interactions between patients and HCWs. This study provided valuable insights into the factors that influenced the experience of the patient and HCW, as well as the effect on quality of care delivered, when their interaction was interrupted by an unplanned phone call.

Professionalism and mutual respect are values that underpin the stakeholders’ response to phone calls that occur during consultations. Stakeholders should uphold these values in any patient- HCW interaction by taking appropriate actions to reduce the likelihood of occurrence of phone calls during consultation and to exercise discretion on phone use in order to minimize disruption of the consultation.

Although patients in this study expressed their acceptance of HCWs attending to phone calls during the consultation, it would be a polite gesture for the HCW to apologize before taking the call and to give a quick explanation of the origin of the call before resuming the consultation.

Safety is a key concern amongst the HCWs in this study. Interruptions during the process of healthcare delivery, compromising patient safety, is emerging as an important area of research. [16] Phone-calls occurring on the job have been described by HCWs in this study to cause task and thought interruptions, and were associated with increased risks of medical errors. The relationship between interruptions and the risk of errors could be better understood by referring to a model first described by Trafton et el [17]. They described the impact of interruptions on the cognitive process. When interruptions occurred, the primary task was disrupted and engagement in the secondary task began. The interruption lag was described as the period where the primary task was halted and the secondary task was initiated. On the other hand, the resumption lag was described as the period where the secondary task was completed and the primary task was resumed. The lack of prospective memory formation during the interruption lag and the lack of environment cues during the resumption lag had been described to impair the ability of the interrupted individual to complete the task accurately [18-21]. In addition, these calls would often require the HCWs to receive new information and engage in other tasks. This “information chaos”, described by Beasley et el [22], is often what primary care physicians have to grapple with. This experience was described by all categories of HCWs who participated in this study.

The timing of the phone call has implications on patient safety. Phone interruptions occurring at task boundaries, described as points in between tasks, have been shown to be less disruptive than if they occurred during task execution [16]. As it would be impossible to limit calls to occur only during task boundaries, HCWs would have to learn to cope by adopting counter-measures such as deliberately slowing down their work processes during the phone interruption. According to Li SY et el, this measure allowed the HCW to form prospective memory of the primary task and re-attend to the primary task with the help of cognitive and environment cues [16]. However, this would be a challenge in the context of a high workload environment, described in our setting.

Phone interruptions also jeopardize the efficiency that is expected of a primary health care system that serves a large number of patients daily. The duration of phone conversation would have down stream effects such as increase wait time for subsequent patients, and as a result affect their experience. HCWs described negative emotions such as stress and frustration; understandably as the phone interruptions affected their performance efficiency in the context of a high workload.

In view of the impact of phone interruptions on patient safety and the mental well-being of the HCWs, it would be important to minimize their occurrence, especially those that are trivial and irrelevant to the consultation. Nonetheless, some phone calls play an important role in alerting HCWs of errors, allowing timely interventions to rectify them. Phone calls of this nature are exceptions to those that would otherwise generate negative sentiments amongst HCWs.

This study alluded to phone interruptions having important consequences such as risk to patient safety and compromise on HCWs’ mental-well-being. It would be important to educate HCWs on ways to minimize the occurrence of phone interruptions, manage these interruptions and their reactions as a result of these interruptions. In addition, HCWs could be taught communication skills to mitigate the experience of the affected patient. Such education could be incorporated into staff orientation programs and would better prepare new staff in managing the interrupted interactions.

Leveraging on information technology, alternative modes of communication such as text messaging via computers or smart phones should be explored and considered for implementation. This mode of asynchronous communication will allow the HCWs to respond at their convenience. The lessened impact on task and thought interruption could have a positive impact on error and stress reduction.

In comparison with HCWs, most patients appeared to be less affected by the phone interruptions. This was also reported by Dearden et al [2], where 65 % of the surveyed patients were unaffected when their consultations were interrupted. Our study revealed that patients generally perceived, that taking phone calls constituted part of the HCW’s professional duties and would tolerate such interruptions. Besides, patient’s satisfaction of the consultation could be influenced by other factors such as wait-time, time spent with the doctor, the doctor-patient communication and relationship, provision of adequate medical information, quality of treatment, and physician characteristics such as their display of empathy and competency [23–25]. It is interesting to note that phone interruptions could potentially affect some of these factors, and remain an important area to address.

This study revealed that patients appeared to be less cognizant than HCWs of the risks associated with phone interruptions. Increasing their awareness of the effect of phone interruptions on patient safety would be an important step towards changing patient’s behavior in managing calls during the consultation. Key messages could be communicated through posters, videos, workshops, and opportunistic education in the event of an interrupted consult. Further research is required to evaluate the effectiveness of such interventions in reducing the occurrence of phone interruptions during consultations, minimizing adverse outcomes and improving the experience of patients and HCWs during their encounter in primary care settings.

### Strengths and limitations of the study

The study gathered perspectives from both the patients and HCWs with regards to their response to a common disruption in the primary care practice. Views of the multi-disciplinary primary healthcare team would be relevant to primary healthcare settings that adopt team-based care delivery. The multi-disciplinary healthcare team appeared to have similar experience with such interruptions, regardless of their primary healthcare role. We included the patients’ perspectives and experience in this study to provide a balanced understanding of the effects on multiple key stakeholders in primary care.

The investigators provided a platform for the patients and HCWs to exchange their views face-to-face during the third FGD. It led to an important observation that safety dominated the discussion amongst the HCWs whilst it was not a key issue for the patients. It was an opportunity to promote mutual understanding of the issues experienced between the stakeholders, and for the patients to appreciate the concerns of the HCWs with regards to patient safety as a result of phone interruptions.

Other forms of interruptions, such as knocks on the door, interruptions by other patients were not included in this study. The study involved participants from a high volume public primary care institution, which may not be relevant to other primary care practices that are of lower patient volume or are from the private sector.

## Conclusion

Professionalism and respect are values that underpin the responses and reactions of HCWs and patients when their consultation is interrupted by unplanned phone calls. Concern about the potential jeopardy on patient safety and reduced work efficiency contributed to the HCWs’ responses to phone interruptions. Patients on the other hand were less affected and were perceived to be less cognizant of the risks associated with these interruptions. Measures such as raising patients’ awareness of the risks, providing relevant training to HCWs to manage these interruptions and utility of alternative forms of communication for less urgent matters amongst HCWs could be introduced to mitigate the adverse impact. The effectiveness of these measures awaits further evaluation in future research.

## Abbreviations

HCWs: Healthcare workers
FGD: Focus group discussion
SHP: SingHealth Polyclinics
CIRB: SingHealth Centralised Institutional Review Board
